# Ethical challenges related to assistive product access for older adults and adults living with a disability: a scoping review protocol

**DOI:** 10.1186/s13643-017-0419-5

**Published:** 2017-02-01

**Authors:** Winnie Sun, Michael G. Wilson, Daphne Schreiber, Rosalie H. Wang

**Affiliations:** 1 Faculty of Health Sciences, University of Ontario Institute of Technology, 2000 Simcoe St. N., Oshawa, ON L1H 7K4 Canada; 2 McMaster Health Forum, McMaster University, 1280 Main St W, MML-417, Hamilton, ON L8S 4K1 Canada; 3 Centre for Health Economics and Policy Analysis, McMaster University, Hamilton, Canada; 4 Department of Health Evidence and Impact, McMaster University, Hamilton, Canada; 5 March of Dimes Canada, 10 Overlea Blvd, Toronto, ON M4H 1A4 Canada; 6 Department of Occupational Science and Occupational Therapy, University of Toronto, 500 University Avenue, Toronto, ON M5G 1V7 Canada

**Keywords:** Scoping review, Assistive technology, Assistive products, Ethics, Technology access, Equity

## Abstract

**Background:**

Despite the surge of research and development in assistive products for older adults and/or people living with disabilities, policies on access and procurement have lagged in responding to the growing demand from users. Developing policies to address these concerns require an understanding of the ethical challenges underlying approaches for providing assistive products. The purpose of this scoping review is to identify and map the literature pertaining to ethical challenges related to assistive product access and procurement to inform policy development.

**Methods/design:**

We will use established approaches to conducting scoping reviews which include five stages: (1) conducting broad searches to identify potentially relevant literature, (2) refining selection criteria, (3) reviewing search results, (4) mapping literature according to conceptual areas of interest, and (5) summarizing results. We will analyze data by thematically grouping the descriptions of assistive products identified in the included articles and conducting a content analysis to iteratively develop a targeted synthesis of literature focused on ethical challenges in relation to assistive product access and procurement by older adults and/or adults living with disabilities.

**Discussion:**

Our scoping review findings will focus on and provide insight about the models, frameworks, and principles that have been used to understand ethical challenges related to technology access and procurement. We will use the findings to help inform a series of citizen panels in Canada to identify Canadians’ values and preferences for enhancing equitable access to assistive products.

**Electronic supplementary material:**

The online version of this article (doi:10.1186/s13643-017-0419-5) contains supplementary material, which is available to authorized users.

## Background

The world’s demographic structure is changing dramatically due to population aging. The number of people who are 60 years old or older is projected to double to over 2 billion people between 2013 and 2050 [1]. The likelihood of having a disability and the severity of disability increases with age [2]. While estimates vary, up to one billion people in the world live with some form of disability and these numbers will continue to increase due to prolonged life spans [3]. In the years ahead, the use of assistive technology will become increasingly important as the prevalence of disability rises. Assistive technology is vital to people with disability in promoting self-management and independence, as well as helping them to perform daily tasks by compensating for physical, sensory, and cognitive impairments [4]. The United Nations Convention on the Rights of Persons with Disabilities also emphasizes the need for countries to ensure that persons with disabilities engage and participate fully in all areas of their daily lives and communities [5]. In defining societal obligations (article 4), the convention explicitly recognizes the important role that technology can play to address issues of basic human rights and fundamental freedoms.

Assistive technology encompasses a broad range of supports with varying terms and definitions used in the research literature, legislation, policy, and practice. A commonly cited definition of assistive technology (used in the Assistive Technology Act of 2004 in the USA) indicates that it is technology that can be used in both assistive technology devices and services [6]. In this case, an assistive technology device includes “any item, piece of equipment, or product system, whether acquired commercially, modified, or customized, that is used to increase, maintain, or improve functional capabilities of individuals with disabilities” and a service includes “any service that directly assists an individual with a disability in the selection, acquisition, or use of an assistive technology device” [6]. The International Organization for Standardization (ISO) in its classification of Assistive Products for Persons with Disability (ISO9999:2016) does not specify assistive technology services but includes “any product (including devices, equipment, instruments, and software), especially produced or generally available, used by or for persons with disability” [7]. The purposes of the included assistive products range from enabling participation; protecting, training, measuring, or substituting for body functions/structures and activities; to preventing impairments and limitations. ISO9999:2016 aimed to harmonize terms used in the World Health Organization (WHO)’s International Classification of Functioning, Disability and Health (ICF) [8]. The ICF includes Products and Technology as one group within its classification of Environmental Factors [9]. Assistive products and technology in the ICF are defined as “any product, instrument, equipment or technology adapted or specially designed for improving the functioning of a disabled person” and do not explicitly include purposes such as prevention of impairments and limitations. Classification is based on functions enabled by the products and technology and includes the following: personal use in daily life; personal indoor and outdoor mobility and transportation; communication; education; employment; culture, recreation, and sport; and practice of religion and spirituality.

In spite of the high need for and use of assistive technology, unmet needs exist for access and procurement [10]. Access is related to the fair and just distribution of resources to ensure individuals have equitable opportunities to obtain appropriate products/services based on their needs [11]. On the other hand, procurement refers to a transparent process that involves the effective delivery of quality product/service to an individual at the right time and place [12]. A review of the literature reveals that significant challenges exist in addressing the human rights of persons with assistive technology needs in relation to access and procurement, as significant disparities in the provision and access to assistive technology programs have been found in several countries [13–15]. For instance, funding and services are highly fragmented with assistive technologies provided through both federal and provincial agencies, non-profit and charitable organizations, and private insurance providers [10]. Policies for access through governmental agencies are regionally defined, and access is often restricted according to age and physical and medical needs [16]. Funding for assistive technology related to cognitive disability and functional and participation needs is particularly difficult to access [17]. Fragmentation and variation in policies and services result in the failure of systems to provide for those who need assistance, to meet our societal obligations for equity of access to assistive technologies and opportunities, and to address economic concerns. Furthermore, despite the surge of research and development in technologies from other sectors, and many promising technologies (including tele-health, tele-homecare, and information and communication technologies that can support the needs of a growing population of users), policy related to access and procurement has lagged in responding to innovations and the growing demand from users [14, 18]. There is a clear urgency for a coordinated research, implementation, and policy response to assistive product access and procurement that is proactive, responsive, and sustainable to match technology advancement. There is also the need to examine the ethical concern of equity in the availability of new assistive products to address the need for the implementation of the human rights of persons with technology needs in our society, including people acquiring disability as they age, aging with a disability, and aging well.

### Objectives

Our objective is to conduct a scoping review to examine the ethical challenges related to assistive product access and procurement. Our specific objectives are to:

1. Identify the challenges related to assistive product access and to examine the models, frameworks, and principles that have been used to understand the ethical dimensions related to these challenges, with a focus on their use for older adults and/or adults living with a disability, and
2. Develop a conceptualization of ethical challenges related to assistive product access and procurement.

## Methods/design

We chose a scoping review as our approach because (to our knowledge) the ethical challenges related to assistive product access and procurement and the implications of these have not been comprehensively reviewed, and an important first step towards such a comprehensive assessment is to systematically map the key concepts, main sources, and types of evidence available to identify gaps in the research area. We will use established systematic and transparent approaches to conducting scoping reviews [19], which include five stages: (1) conducting broad searches to identify potentially relevant literature, (2) refining selection criteria, (3) reviewing search results, (4) mapping literature according to conceptual areas of interest, and (5) summarizing results. This protocol is not registered with PROSPERO, which does not provide registration of scoping review protocols.

### Literature searches

Our search strategy will involve identifying published journal articles and gray literature to ensure that we cover the breadth and comprehensiveness of the available literature addressing the ethical implications of assistive product access. We will search the following 22 databases to identify relevant literature: The Cochrane Library (including Cochrane Database of Systematic Reviews and reviews indexed in the Database of Abstracts of Reviews of Effects), MEDLINE, Health Star, PsycINFO, EMBASE, AMED (Allied and Complementary Medicine), International Political Science Abstracts, Social Work Abstracts, Joanna Briggs Institute EBP database, CINAHL, AgeLine and Social Science Abstracts (EBSCO), Applied Social Sciences Index and Abstracts (ASSIA), ProQuest Worldwide Political Science Abstracts, Social Services Abstracts, Sociological Abstracts, Philosopher’s Index, EconLit, Web of Science Core Collection, Scopus, PubMed for non-MEDLINE records, McMaster Optimal Aging Portal (repository of systematic reviews and primary research related to optimal aging that draws on McMaster PLUS, HealthEvidence, Health Systems Evidence).

To identify gray literature, we will search OpenGrey and Grey Literature Report and conduct targeted website searches of government departments and stakeholder organizations, such as the Medical Devices Publications, Canadian Disability Policy Alliance, Canadian Agencies for Drugs and Technologies in Health (CADTH), UN ENABLE (Convention on Rights of Persons with Disability), International Federation on Aging, and World Health Organization (WHO). We will supplement our website searches by contacting key informants from organizations within national and international settings, including AGE-WELL NCE (Aging Gracefully across Environments using Technology to Support Wellness, Engagement and Long Life Networks of Centres of Excellence Inc.) in Canada and the Bridging Aging and Disability International Network. We will also scan the reference lists of articles that we included in the final scoping review as a final check for any articles we may have missed.

We will limit our searches to English only, but the date range of our searches will not be limited to ensure that we cover the breadth and comprehensiveness of the available literature. We will search the databases listed above using the following combination of terms: (ethic* OR equit* OR equal* OR fair* OR disparit* OR distributi*) AND (“assistive technology” OR “assistive technologies” OR “assistive device” OR “assistive devices” OR “adaptive device” OR “adaptive devices” OR “adaptive technology” OR “adaptive technologies” OR “rehabilitation device” OR “rehabilitation devices” OR “rehabilitation technology” OR “rehabilitation technologies” OR “assistive product” OR “assistive products”). The search strategy and the number of results from each of the databases are outlined in Additional file 1.

### Selection criteria development

We will include empirical and non-empirical documents that focus on the ethical challenges related to access to assistive products for adult populations (18 years of age and older) with disabilities or health conditions. For this review, we will use an existing definition for assistive product as defined by ISO9999:2016 which describes it as “any product (including devices, equipment, instruments and software), especially produced or generally available, used by or for persons with disability” [7]. Examples of these products may include wheelchairs, communication devices, bath aids, environmental sensors, and assistive software.

### Reviewing search results

All search results will be reviewed independently by two reviewers using the selection criteria, and records will be classified as “potentially relevant” or “exclude.” In the event of a discrepancy between the two reviewers, the piece of literature will be classified as “potentially relevant” at this stage. The full text of “potentially relevant” articles will be retrieved and two reviewers will again independently review the texts to make a final assessment for inclusion in the scoping review. When consensus on whether a document should be included cannot be reached during the full-text review, a final decision will be made by a third reviewer. Reference manager software, Mendeley, and reviewing manager software, Covidence, will be used to facilitate the management of references and the reviewing process.

### Conceptual mapping

We will conceptually map the papers that fit our selection criteria using an iterative process. The purpose of conceptual mapping in scoping reviews is to categorize relevant papers into domains and topics of interest to identify areas that are conceptually rich and areas where there appear to be conceptual gaps [20]. We will start by having 10 of the included papers independently assessed by two reviewers using the draft conceptual mapping form (coding framework) provided in Additional file 2. Following this assessment, we will meet as a team to revise the coding framework to add, remove, or reorganize parts of the framework based on our experience applying it to the initial sample of papers. An additional 10 papers will then be reviewed independently by two researchers using this coding framework. The two researchers will then compare their coding of these 10 papers to ensure they are coding reliably, and the team will make any additional needed revisions to the framework. The final coding framework will then be used to code all of the included papers (including 20 coded in the pilot process) by one of these two researchers.

Our initial framework includes six domains: (1) type of document (research and non-research); (2) population characteristics (e.g., age of population of focus, disabilities or health conditions, and sociocultural characteristics); (3) context (country or region focus, type of provider involved, and sector involved); (4) type of assistive product; (5) assistive product access and procurement; and (6) ethical concepts (autonomy, beneficence, non-maleficence, and justice). Other relevant ethical concerns include equitable access, fairness, disparities, distributive justice, social justice, advocacy, resource allocation, and ageism. In addition to the categories included in the conceptual mapping, the reviewers will assess each paper by identifying those that are likely to offer important insights into the ethical challenges that can help to inform discussions about equitable access to assistive products.

### Summarizing results

The presentation of the scoping review results will include an outline of how many articles are identified and selected, which will include a narrative description of the search decision process accompanied by the search decision flowchart using the PRISMA (Preferred Reporting Items for Systematic Reviews and Meta-Analyses) reporting guideline [21]. A copy of the PRISMA Protocol reporting guidelines is outlined in Additional file 3.

We will summarize the conceptual mapping results in tabular format. Specifically, we will summarize the number of papers in each of the conceptual mapping domains both overall and according to the two populations of interest (older adults and adults living with a disability). We will use these findings to identify areas of conceptual richness and where there are gaps. In particular, we will derive potential areas for future in-depth synthesis (e.g., using literature related to specific ethical challenges for each of the populations of interest).

## Discussion

Underlying our scoping review approach is an emphasis on systematically and transparently mapping the literature to better understand the ethical challenges related to assistive product access and procurement. We will disseminate our scoping review findings by summarizing the key implications and lessons learned about the ethical considerations for addressing barriers to assistive product access and procurement. Our scoping review will help inform future work towards developing ethical decision-making frameworks and models that are useful for policymakers, stakeholders, and researchers in addressing equitable access and procurement of assistive products.

One component of our knowledge translation plan is to disseminate the findings through at least one conference presentation and to submit at least one manuscript to an open-access, peer-reviewed journal. Another component of our knowledge translation activities is to utilize our scoping review findings to prepare a plain language brief (called a “citizen brief”) that will serve as key input for citizen panels that we plan to convene in Canada [22]. The panels will identify citizen values and preferences for (1) improving access to assistive products that enable health, participation, and well-being in ways that align with their values and (2) establishing a proactive, sustainable, and responsive approach to ensuring access to match the pace of rapidly advancing technologies. Findings from the citizen panels will be used to contribute to the development of a more detailed evidence brief that will be used to inform a stakeholder dialogue with key leaders and decision-makers (policymakers, stakeholders, and researchers) in Canada [23]. The evidence brief will mobilize the best available evidence from the scoping review findings to frame the factors contributing to the problem related to assistive product access and identify policy options to address the problem and implementation considerations to address these challenges. This approach on stakeholder engagement will help mobilize our scoping review findings to inform policy and practice in Canada.

## Additional files

Additional file 1: Literature search strategy. (DOCX 18 kb)
Additional file 2: Conceptual mapping form. (DOCX 22 kb)
Additional file 3: PRISMA Protocol checklist. (DOCX 26 kb)

## Abbreviations

AGE-WELL NCE: Aging Gracefully across Environments using Technology to Support Wellness, Engagement and Long Life Networks of Centres of Excellence Inc.
CADTH: Canadian Agencies for Drugs and Technologies in Health
ICF: International Classification of Functioning, Disability and Health
ISO: International Organization for Standardization
PRISMA: Preferred Reporting Items for Systematic Reviews and Meta-Analyses
UN ENABLE: Convention on Rights of Persons with Disability
WHO: World Health Organization
