# Supplementary material for: Ethical challenges related to assistive product access for older adults and adults living with a disability: a scoping review protocol
Source: Syst Rev. 2017 Feb 1;6:24. doi: 10.1186/s13643-017-0419-5 (PMC5286736; doi:10.1186/s13643-017-0419-5)
Supplement: Additional file 2: — Conceptual mapping form. (DOCX 22 kb) [file 13643_2017_419_MOESM2_ESM.docx]

**Additional File 2: Conceptual mapping form**

| **Domains** | **Categories** |
| --- | --- |
| **Title** | - Include complete reference (i.e. authors, publication date) |
| **Academic discipline** | - e.g. health services, biomedical, social science, psychology, systems and policy, economics, health technology assessment |
| **Type of document** | **Research**   - Systematic review - Primary research   - RCT   - Observational     - Cohort     - Cross-sectional   - Qualitative     - Case study   - Mixed methods   **Non-research**   - Review (not systematic) - Theory - Editorial - Other:   - Situation analysis   - Toolkit   - Options framing   - Guidance   - Stakeholder position paper   - World Health Assembly (WHA) resolution |
| **Population characteristics** | - **Age**   - Adults (between 18 and 64)   - Older adults (65 and older) - **Disabilities or health conditions**   - Primarily physical disability according to WHO World Report on Disability [3]     - Sensory functions and pain     - Voice and speech functions     - Functions of the cardiovascular, haematological, immunological and respiratory systems     - Functions of the digestive, metabolic, endocrine systems     - Genitourinary and reproductive functions     - Neuromusculoskeletal and movement-related functions     - Functions of the skin and related structures   - Primarily cognitive/learning disability   - Disability related to a mental health condition   - Disability related to multimorbidity (living with three or more chronic diseases) - **Socio-cultural characteristics**   - Location of residence     - Living at home     - Living in a senior’s residence (i.e., some supports provided, but not institutional care)     - Living in a long-term care setting     - Living in a rural or remote setting     - Homeless or marginally housed   - Low socioeconomic status   - Indigenous populations |
| **Context** | - **Country focus**   - WHO region     - African region     - Americas region     - Eastern Mediterranean region     - South East Asia region     - Western Pacific region     - European region   - Income level (based on World Bank classification [23])     - Low-income country     - Middle-income country     - High-income country - **Type of provider(s) involved**   - Physician   - Generalist   - Specialist   - Geriatrician   - Nurse   - Nurse practitioner   - Pharmacist   - Allied health professional   - Lay/community health worker   - Caregivers (i.e., unpaid caregivers, informal caregivers, family caregivers, etc.) - **Type of sector (s) involved:**   - Primary care   - Home care   - Hospital care   - Rehabilitation   - Long-term care   - Public health |
| **Type of assistive product** | **Type according to purpose defined by ISO9999:2016 [6]:**   - For participation - To protect, support, train, measure or substitute for body functions/structures and activities - To prevent [impairments](https://www.iso.org/obp/ui/#iso:std:iso:9999:ed-6:v1:en:term:2.11), [activity limitations](https://www.iso.org/obp/ui/#iso:std:iso:9999:ed-6:v1:en:term:2.2)or participation restrictions   **Type according to function as defined by International Classification of Functioning, Disability and Health (ICF, World Health Organization) [7] and ISO9999:2016 [8]:**     - For personal use in daily living - For personal indoor and outdoor mobility and transportation - For communication - For education - For employment - For culture, recreation and sport - For practice of religion and spirituality |
| **Technology Access and Procurement** | - Limitations and challenges related to access, services, funding, policy, regulation and legislations - Facilitators and barriers to technology access and procurement |
| **Ethical concepts and challenges** | **Based on Beauchamp and Childress (2009) [24]:**   - **Autonomy** (preserving the rights of individuals to make decisions about their own lives and the right to privacy) - **Beneficence** (The shared responsibilities and positive actions we have for each other, doing something good, and preventing what is harmful) - **Non-maleficence** (Exercising due care so as not to unintentionally harm others through their actions) - **Justice (**A fair distribution of benefits and burdens)   **Additional relevant ethical concerns:**   - Equity - Equality - Fairness - Disparities - Distributive justice - Social justice - Advocacy - Resource allocation - Ageism |

*Categories to be iteratively revised and supplemented throughout the title and abstract review phase.
