# Supplementary material for: Ethical challenges related to assistive product access for older adults and adults living with a disability: a scoping review protocol
Source: Syst Rev. 2017 Feb 1;6:24. doi: 10.1186/s13643-017-0419-5 (PMC5286736; doi:10.1186/s13643-017-0419-5)
Supplement: Additional file 3: — PRISMA Protocol checklist. (DOCX 26 kb) [file 13643_2017_419_MOESM3_ESM.docx]

**Additional File 3: PRISMA-Protocol Checklist**

| **Section and Topic** | **Item No.** | **Checklist item** |
| --- | --- | --- |
| **ADMINISTRATIVE INFORMATION** | | |
| **Title:**   - Identification - Update | 1a  1b | - This report is a scoping review protocol - This is a new protocol submission |
| **Registration** | 2 | - N/A |
| **Authors:**   - Contact - Contributions | 3a  3b | - **Winnie Sun, RN, PhD**   University of Ontario Institute of Technology  2000 Simcoe St. N.  Oshawa, ON L1H 7K4  Email: [winnie.sun@uoit.ca](mailto:winnie.sun@uoit.ca)   - **Michael Wilson, PhD**   McMaster University  1280 Main St. West, MML-417  Hamilton, ON L8S 4K1  Email: [wilsom2@mcmaster.ca](mailto:wilsom2@mcmaster.ca)   - **Daphne Schreiber, MSc**   March of Dimes Canada  10 Overlea Blvd.  Toronto, ON M4H 1A4  Email: [dschreiber@marchofdimes.ca](mailto:dschreiber@marchofdimes.ca)   - **Rosalie Wang, PhD, OT Reg. (Ont.)**   Occupational Science and Occupational Therapy, University of Toronto  160 - 500 University Avenue, Toronto, ON, M5G 1V7  Email: [rosalie.wang@utoronto.ca](mailto:rosalie.wang@utoronto.ca)   - All authors (WS, MW, DS and RW) provided input into the development of the scoping review protocol, and have read and approved this manuscript. |
| - Amendments | 4 | - N/A |
| **Support:**   - Sources - Sponsor - Role of Sponsor or Funder | 5a  5b  5c | - This project is funded by AGE-WELL NCE (Aging Gracefully across Environments using Technology to Support Wellness, Engagement, and Long Life, Networks of Centres of Excellence). - In-kind contributions from March of Dimes Canada - Funder or sponsor has no role in developing the protocol. |
| **INTRODUCTION** | | |
| **Rationale** | 6 | - Despite the surge of research and development in assistive products, policies on access and procurement have lagged in responding to the growing demand from users. Developing policy to address this gap requires an understanding of the ethical challenges underlying approaches for providing assistive products. The purpose of this scoping review is to systematically and transparently identify the literature about the ethical concepts and challenges related to assistive product access and procurement to inform policy development. |
| **Objectives** | 7 | - Identify the challenges related to assistive product access and to examine the models, frameworks and principles that have been used to understand the ethical dimensions related to these challenges, with a focus on their use for older adults and/or adults living with a disability, and - Develop a conceptualization of ethical challenges related to assistive product access and procurement. |
| **METHODS** | | |
| **Eligibility criteria** | 8 | - Limit to English only, but date range of searches will not be limited.   **Population of focus**:   - Adult populations who are 18 years of age and older, with emphasis on older adults (65 years of age and older), who are living with any types of disability and heath conditions   **Concepts:**   - Assistive products and ethical issues related to access and procurement are the primary concepts. - Models, frameworks and principles that have been used to understand ethical issues and any challenges related to access to and procurement of assistive products. - Inclusion criteria for assistive product: “any product (including devices, equipment, instruments and software), especially produced or generally available, used by or for persons with disability” [6] - May include products used for the purposes of participation; protection, support, training, measuring or substituting for body functions/structures and activities; or prevention of [impairments](https://www.iso.org/obp/ui/#iso:std:iso:9999:ed-6:v1:en:term:2.11), [activity limitations](https://www.iso.org/obp/ui/#iso:std:iso:9999:ed-6:v1:en:term:2.2)or participation restrictions. [6]. - May be categorized according to function: personal use in daily living; personal indoor and outdoor mobility and transportation; communication; education; employment; culture, recreation and sport; or practice of religion and spirituality [7]   **Context:**   - All types of health care settings such as primary care, home care, hospital care, rehabilitation, long-term care and public health. - Articles from different countries worldwide |
| **Information sources** | 9 | - Published and grey literature addressing the ethical implications about assistive product access. - Primary research studies, systematic reviews (e.g. meta-analyses), concept papers, white papers, policy documents and guidelines. - 22 databases included: The Cochrane Library (including Cochrane Database of Systematic Reviews and reviews indexed in the Database of Abstracts of Reviews of Effects); MEDLINE; EMBASE; HealthStar; PsycINFO; International Political Science Abstract; AMED (Complementary Medicine: Social Work Abstracts; Joanna Briggs Institute EBP database; CINAHL; Social Science Abstracts; (EBSCO); AGELINE; Applied Social Sciences Index and Abstracts (ASSIA); ProQuest Worldwide Political Science Abstracts; Social Services Abstracts; Philosopher’s Index; Sociological Abstracts; EconLit; Web of Science Core Collection; Scopus; PubMed for non-MEDLINE records; McMaster Optimal Aging Portal; HealthEvidence.org (for reviews related to public health); and Health Systems Evidence. - Grey literature including Open Grey and Grey Literature Report; targeted searches of websites and resources. - Supplement website searches by contacting key informants from organizations within national and international settings. - Hand-searching the reference lists of those included data sources. |
| **Search strategy** | 10 | - A sample draft of search strategy is included in Additional File 1. |
| **Study records:**   - Data management - Selection process - Data items | 11a  11b  12 | - Study records will be managed by reference manager “Mendeley” and by review manager “Covidence”. - All search results will be reviewed in duplicate by two independent reviewers using the selection criteria and records will be classified as ‘potentially relevant’ or ‘exclude’ The full-text of ‘potentially relevant’ articles will be retrieved and two reviewers will make a final assessment for inclusion in the scoping review. When consensus on whether a document should be included cannot be reached during the full-text review, a final decision will be made by a third reviewer. - Data variables are listed in Additional File 2 Conceptual Mapping Form |
| **Outcomes and Prioritization** | 13 | - Primary outcomes are ethical challenges related to assistive technology access and procurement.   Types of ethical principles examined (Beauchamp & Childress, 2009 [24]):   - Autonomy - Beneficence - Non-maleficence - Justice |
| **Risk of bias in individual studies** | 14 | - N/A |
| **Data synthesis** | 15a  15b | - Study data will not be quantitatively synthesized - Data synthesis will involve the identification of emerging themes and concepts based on the summaries and data extracted from each article. The results from the conceptual mapping will be used to identify areas of conceptual richness, and the data synthesis will be supplemented with the detailed data extraction table to produce a distillation of key messages. - Data synthesis will include a summary of the conceptual mapping result in tabular format. A summary of the number of papers in each of the conceptual mapping domains both overall and according to the two populations of interest (older adults and adults living with a disability) will be included. These findings will be used to identify areas of conceptual richness and where there are gaps. In particular, areas for potential areas for future in-depth synthesis (e.g., using literature related to specific ethical concepts and challenges for each of the populations of interest) will be derived from the data synthesis. |
| **Meta-biases** | 16 | - N/A |
| **Confidence in cumulative evidence** | 17 | - N/A |
